# Community Attitude and Associated Factors towards People with Mental Illness among Residents of Worabe Town, Silte Zone, Southern Nation’s Nationalities and People’s Region, Ethiopia

**DOI:** 10.1371/journal.pone.0149429

**Published:** 2016-03-01

**Authors:** Asres Bedaso, Tebikew Yeneabat, Zegeye Yohannis, Kufa Bedasso, Fetuma Feyera

**Affiliations:** 1 Department of Psychiatry, College of Health and Medical Science, Hawassa University, Hawassa, Ethiopia; 2 Department of Midwifery, College of Medical and Health Sciences, Debre Markos University, Debre Markos, Ethiopia; 3 Department Psychiatry, Amanuel Mental Specialized Hospital, Addis Ababa, Ethiopia; 4 Department of Nursing, College of Medicine and Health Sciences, Minilik II Referral Hospital, Addis Ababa, Ethiopia; 5 Department of Nursing, College of Medical and Health Sciences, Debre Markos University, Debre Markos, Ethiopia; Örebro University, SWEDEN

## Abstract

**Background:**

Mental illnesses worldwide are accompanied by another pandemic, that of stigma and discrimination. Public understanding about mental illnesses and attitudes towards people with mental illness play a paramount role in the prevention and treatment of mental illness and the rehabilitation of people with mental illness.

**Objective:**

To assess community attitude and associated factors towards people with mental illness.

**Methods:**

Community based cross-sectional study was conducted from April 28 to May 28, 2014. Quantitative data were collected through interview from 435 adults selected using simple random sampling. Data were collected using community attitude towards mentally ill (CAMI) tool to assess community attitude towards people with mental illness and associated factors. Multiple linear regression analysis was performed to identify predictors of community attitude towards people with mental illness and the level of significance association was determined by beta with 95% confidence interval and P less than 0.05.

**Results:**

The highest mean score was on social restrictiveness subscale (31.55±5.62). Farmers had more socially restrictive view (β = 0.291, CI [0.09, 0.49]) and have less humanistic view towards mentally ill (β = 0.193, CI [-0.36, -0.03]). Having mental health information had significantly less socially restrictive (β = -0.59, CI [-1.13, -0.05]) and less authoritarian (β = -0.10, CI [-1.11, -0.06]) view towards mentally ill but respondents who are at university or college level reported to be more socially restrictive (β = 0.298, CI [0.059, 0.54]). Respondents whose age is above 48 years old had significantly less view of community mental health ideology (β = -0.59, CI [-1.09, -0.08]).

**Conclusion and Recommendation:**

Residents of Worabe town were highly socially restrictive but less authoritarian. There was high level of negative attitude towards people with mental illness along all the subscales with relative variation indicating a need to develop strategies to change negative attitude attached to mental illness in Worabe town at community level.

## Background

Globally, individuals with mental illnesses are among the most vulnerable and the poorest, and the discrimination they experience is unacceptable and a cause for international indignity [1]. Mental illness can affect anyone, in any age, gender, culture, ethnicity, or social class. Regardless of who they are, people who have been diagnosed with a mental illness are all likely to experience stigma, and despite this fact, mental health has been hidden behind a curtain of stigma and discrimination for very long period [2–4].

The stigma associated with psychiatric disease has been shown to be widespread and continues to be a barrier to rehabilitation for many people in our society. People stigmatized because of mental illness are a group who are wrongfully shamed, humiliated and marginalized [5]. Bohner and Dickel define an attitude as “evaluation of an object of thought ranging from the mundane (ordinary) to the abstract, including things, people, groups, and ideas” [6]. Negative attitudes by the majority of people (‘the public’) toward individuals or characteristics of individuals give rise to stigma and can lead to discriminatory (potentially illegal) behavior [7,8].

Stigma of mental ill health is more restricting than the illness itself. Research has shown that people with mental health problems are prejudiced, find it hard to get jobs and sustain friendships and relationships. Most people have little knowledge about mental illness and their opinions are often factually incorrect [9]. Consequently, it generates poor self-esteem, low self-confidence and reduced motivation, threat to job opportunities and result in isolation of the individual with mental illness [1]. It is not only a consequence of mental illness but also a factor that interferes with help-seeking behavior, and it may delay treatment-seeking in patients with mental illness [10].

Community’s perspectives and attitude towards people with mental illness (PWMI) play a paramount role in mental health care. Community members can act as reinforcing agents for preventive, treatment seeking and drug compliance behaviors. In developing countries like Ethiopia, where mental health services are limited or too scarce and PWMI often delay seeking treatment for their mental illness, the community plays an essential role in the treatment and rehabilitation of patients with mental illness. However, community members commonly play a negative role and worsen the consequences of mental illness among patients [11].

Studies done in different areas have shown stigma towards mentally ill individuals is mainly deep rooted with various sociodemographic factors [11–20].

In Ethiopia there are few published studies [11,14] assessing community attitude towards people with mental illness and no studies done in Worabe town concerning this topic. Therefore, this study have great value on assessing the attitude of the community towards mental illness and hence, the level of negative attitude among the inhabitants of Worabe town.

## Methods

### Study design and setting

Community based cross-sectional study design that employed both quantitative and qualitative methods were conducted from April 28 to May 28, 2014 in Worabe town of Silte Zone, Ethiopia. Worabe town is located 172Km away from Addis Ababa in Southwest direction. According to the 2007 National Housing and Population Census, the projected population of Worabe town for the year 2014/15 was about 15,920 and the estimated number of households was 3249 [21].

### Sample size and sampling procedure

Sample size was calculated using formula for single population mean by taking the following assumptions.

*Z* = standard normal distribution with confidence interval of 95% = 1.96

σ = standard deviation of Community attitude towards mental illness (CAMI) sub scales from study conducted at Gilgel Gibe Field Research Center (GGFRC) [11].

d = absolute precision or tolerable margin of error of 0.05

n = sample size (Table 1).

**Table 1:** Sample size estimation using the mean for CAMI sub scales, Worabe town, Silte zone, SNNPR, Ethiopia, 2014.

Variables	σ	Calculated sample size
Authoritarianism (AU)	0.36	198
Benevolence (BE)	0.39	223
Social restrictiveness (SR)	0.52	415
Community mental health ideology (CMHI)	0.50	384

Finally the mean that give the greatest sample size was selected (σ = 0.52). Therefore,

$$n=\frac{{\left(Z\frac{\alpha }{2}\right)}^{2}\left(\sigma^{2}\right)}{d^{2}}$$

$$n=\frac{{\left(1.96\right)}^{2}{\left(0.52\right)}^{2}}{0.05^{2}}\approx \underline{415}$$

With 5% non-response rate the total sample size was calculated as **435**.

Sampling frame for this study was households from where study subjects were selected. According to the town administration report, the town has two Kebeles and a total of 15920 households. First, households (primary sampling unit) were selected by simple random sampling. Then study subjects from the selected households were selected by simple random sampling. To increase the representativeness of data to the household members, study subjects were heads of households assuming they can represent their family’s thoughts and ideas on the topic. However, individuals aged 18 years and above were included in the study by lottery method in case of absence of households during data collection.

Two focus group discussions (FGDs) were conducted to collect qualitative data. Participants for FGD, who were not included in quantitative study, were selected purposively from kebele leaders, religious leaders, health development army members and health professions.

All residents who were 18 years old and above living for at least 6 months in Worabe town during the time of the study were interviewed and those who were with hearing impairment and unable to respond, and those with severe cognitive impairment were excluded.

### Operational definition

**Negative attitude**: is the discrediting reaction that the general population has, towards mental illness or persons with mental illness.

**Magnitude of negative attitude**: is expressed by each sub scale mean score; the higher the mean score the higher the negative attitude against people with mental illness.

**Authoritarianism**: refers to a view of a person with mental illness as someone who is inferior and requires supervision.

**Benevolence**: refers to humanistic and sympathetic view towards persons with mental illness.

**Social Restrictiveness**: corresponds to the belief that persons with mental illness are a threat to society and should be avoided.

**Community Mental Health Ideology**: refers to the acceptance of mental health services and the integration of persons with mental illness in the community.

**Income**:< 1.25 USD per day (20*1.25*30 = 750ETB per month) under extreme poverty and <2 USD per day (20*2*30 = 1200ETB per month) under poverty [22].

### Data collection procedure and measurements

Quantitative data were collected through interview using CAMI tool. Four Diploma Nurses were recruited as data collectors based on their ability to speak Amharic languages, and having previous experience of data collection. One BSc Nurse was recruited as supervisor. Two days intensive training on tools and data collection techniques was given for data collectors.

Qualitative data were collected by principal investigator through FGD by exploring probing-questions.

The tool consisted of two sets, socio-demographic variables and variables to measure community attitude towards mentally ill patients. The tool included 40 items rated on a five-point Likert scale from 1 to 5 (1 = strongly agree, 2 = agree, 3 = neutral, 4 = disagree, and 5 = strongly disagree). The tool has been previously pretested and validated and the values of reliability index for CAMI sub scales measured using Cronbach alpha was (AU = 0.68, BE = 0.76, SR = 0.80, CMHI = 0.88) [11,13,16,18]. The tool was first developed in English language and translated to Amharic language then back to English language to check consistency. Amharic version questionnaire was used to collect data.

Pretest was done in 5% of the study subjects selected from the areas that were not included in the actual data collection then it was analyzed to check the internal consistency. The reliability of the tool was as found as (AU, α = 0.61, BE, α = 0.6, SR, α = 0.7, CMHI, α = 0.82 and when all 40 items were considered the overall reliability of CAMI scale was α = 0.84).

Interview guide questions containing the community attitude towards persons with mental illness (PWMI) and factors affecting community attitude towards PWMI, and ways to promote community attitude in the area were used to collect qualitative data through FGD.

### Data processing and analysis

Data were coded, edited, cleaned and entered into Epi-info version 7 then exported to SPSS version 20 for analysis.

Independent sample t-test and one way ANOVA were used to determine association of categorical variables with the outcome variables. Finally, variables found to be associated in t-test and one way ANOVA were entered in multiple linear regressions to control confounders. To identify independent factors separate linear regression models were performed to each subscale for variables which showed significant association during t-test (to analyze mean difference between two groups) and one way ANOVA (to analyze mean difference among more than two groups). Significance level between variables and CAMI sub scales were determined by β at P<0.05.

Qualitative data were transcribed to Amharic and translated to English and the main responses were categorized into themes. Thematic areas were identified based on the objective of the study. Different ideas were organized under the themes using an open color coding. The ideas were organized into concepts and presented in narratives using transcribed form of the study participant’s explanation. Finally the narrative qualitative information was triangulated with quantitative finding.

### Ethical consideration

Ethical clearance was obtained from ethical review board of University of Gondar, College of Medicine and Health Science institute of Public Health and Amanuel Mental Specialized Hospital after submission of study protocol for ethical review. Ethical review board approved the methods of data collection and forms of consent. Written consent was sought from Worabe administrative town health office. Since the study included literate and illiterate subjects, informed verbal consent was obtained from each respondent. Each questionnaire was initially prepared with the written consent attached with it for the interviewer to offer the consent for the respondents verbally and participants willing to be interviewed had signed with finger prints and finally the obtained consents were kept with the questionnaire. The respondents were informed that their inclusion in the study is voluntary and they are free to withdraw from the study if they are not willing to participate. Anonymity was maintained to ensure confidentiality of respondents.

## Results

Initially this study proposed to interview 435 individuals aged 18 years and above. But, data collected only from 409 study subjects were included in the analysis making the response rate 94%. The remaining questionnaires from 26 participants were not included in the analysis due to incomplete response.

### Socio-demographic characteristics

About half (50.2%) of the respondents were females. The mean (±SD) age of the respondents was 33 (±10.25) years. More than two third (67.9%) were married and 27.7% were single. The major ethnic group was Silte (81.6%) followed by Gurage 5.4%. Greater proportion of the respondents were Muslim in religion (77.9%) followed by Orthodox Tewahido (10%). About one third (31.1%) attended primary school and 22.3% attended secondary education. Relatively the predominant occupations were housewives (21.1%) and merchants (21.1%). The median (±SD) estimated monthly household income was 800(±601) ETB (S1 Table).

### Mental health information

About half (50.7%) of the study subjects reported that they had information about mental illness. They source of information was from multiple sources (47%), from TV only (23%), from health institutions only (15%) and from magazines only (2%).

### Contact to a person with mental illness, and history of mental illness

More than two third (71.6%) had history of contact with a person with mental illness and 12% of them also had ever have mental illness. The person with mental illness they ever had contact was street resident (47%), family member (24%), neighbor (19%) and friend (10%).

Of those who ever have contact history with persons with mental illness, 26.2% were threatened or hurt and as well 55.4% of residents witnessed others being threatened or hurt.

### Community Attitude towards People with Mental Illness (CAMI)

The highest and lowest mean scores were measured on social restrictiveness (31.5±5.6) and authoritarian (28.9±4.7) respectively (Table 2).

**Table 2:** Mean score and standard deviation of the CAMI subscales, Worabe town, Silte zone, SNNPR, Ethiopia, 2014.

Sub scales	Mean	SD	Cron.alpha
**Authoritarianism**	28.95	4.67	0.61
**Benevolence**	30.57	4.79	0.60
**Social restrictiveness**	31.55	5.62	0.70
**Community mental health ideology**	29.82	6.19	0.82

### Factors associated with CAMI

Community attitude towards people with mental illness, measured by CAMI tool, varies with socio demographic characteristics and information about issues related to exposure to mental health.

Simple linear regression model showed that sex, mental health information, involvement in caring for a person with mental illness, threatened or hurt by a person with mental illness, facing anyone being threatened or attacked by a person with mental illness were associated with authoritarianism and social restrictiveness. Religion and educational status were associated with benevolence and social restrictiveness. Occupational status was associated with benevolence, social restrictiveness and community mental health ideology. Suffering from any mental illness was associated with social restrictiveness and community mental health ideology. Age, ethnicity and monthly estimated household income were associated only with community mental health ideology, social restrictiveness and benevolence respectively.

Sex, educational status, educational status, occupation, monthly estimated household income, mental health information, caring for PWMI, threatened by PWMI, face any one being threatened by PWMI showed statistical significant mean difference in overall CAMI score (Table 3).

**Table 3:** Attitude mean score differences based on socio-demographic backgrounds and issues related to exposure to mental health in Worabe town, Silte zone, SNNPR, Ethiopia, 2014.

Variables	AU		BE		SR		CMHI		Over all CAMI	
	M±SD	P	M±SD	P	M±SD	P	M±SD	P	M±SD	P
**Sex**										
Male	28.3 ± 4.9	0.003	30.3±4.7	0.21	30.5±6.3	0.000	29.3 ± 7.3	0.13	118.4±23.2	0.000
Female	29.6 ± 4.3		31±4.9		32.6±5.2		30.3± 4.9		123.5±19.3	
**Age**										
18–27	28.4 ± 4.7	0.19	30.2 ± 5	0.18	31.9± 5.8	0.45	29.6 ±5.8	0.05	120±15.7	0.67
28–37	29.3± 4.7		30.2± 4.9		31.5± 5.8		29.5± 5.6		120±14	
38–47	28.8± 4.5		31.2± 4.5		30.8±5.4		31.2± 6.5		122±12.2	
≥48	30.1± 4.3		31.9 ± 3		32.1±3.2		28 ± 8.3		122.8±12.8	
**Marital status**										
Married	28.9 ± 4.6	0.66	30.7 ±4.5	0.69	31.9± 5.6	0.199	30.1± 6.4	0.63	120.6±21.1	0.48
Single	29.1± 5.1		30.1 ±5.4		30.8 ± 6.6		29.2 ± 5.9		119.2±23	
Divorced	30.2 ± 3.6		30.9±5.1		29.4 ± 2.9		29.8 ± 5.1		120.3±16.7	
Widowed	27.4 ± 3.2		31.4 ±3.7		30.9 ± 6.2		28.6 ± 5.7		118.3±18.8	
**Ethnicity**										
Silte	28.8 ± 4.7	0.62	30.4 ±4.7	0.33	31.2 ±5.7	0.03	29.8 ± 6.4	0.72	120.2±21.5	0.13
Gurage	28.5 ± 5.8		30.5 ± 6		30.8±7.2		30.6 ± 6.3		120.4±25.3	
Oromo	30 ± 3.8		33.2 ±4.6		34.3±7.3		31.9 ± 5.1		129.4±20.8	
Amhara	30.4± 2.2		31 ± 6.7		31.5 ± 6.0		28.2 ± 4.1		121.1±19	
Tigre	31 ± 1.7		32 ± 5.3		33.2 ± 3.1		30 ± 2.7		126.2±12.8	
Others	29.5 ± 4.7		32 ± 3.7		35 ± 5.0		29.4 ± 5.8		125.9±19.2	
**Religion**										
Muslim	28.8 ± 4.7	0.22	30.2±4.6	0.02	31.1 ± 57	0.002	29.8 ± 6.4	0.98	119.9±21.4	0.71
Orthodox	28.6 ± 4.8		31.3±5.9		31 ± 6.4		30 ± 6.3		120.9±12.7	
Protestant	29.6 ± 3.9		32.5±4.7		34.2 ± 6		29.4 ± 4.8		125.7±19.4	
Catholic	28.9± 2.8		33.7± 4.8		35.9 ±4.7		29.6 ± 6.8		128.1±19.1	
Others	31.3 ± 5.1		32.1±4.1		34.5± 5.6		30.5 ± 4.5		128.4±19.3	
**Education**										
Unable to read & write	29.3 ± 4.8	0.91	30.2±4.4	0.02	31 ± 5.4	0.000	28.8± 6.3	0.17	119.3±20.9	0.034
Able to read & write	28.6 ± 5.1		30.4±4.9		31 ± 6.6		29 ± 6.3		119±22.9	
Primary	28.9 ± 4.9		29.9±5.1		31.1 ±5.5		30.1± 6.4		120± 21.9	
Secondary	28.8 ± 4.2		30.7± 4.5		31.5 ±5.9		30.9± 5.4		121.9± 20	
College/university	29 ± 4.3		32.7± 4.6		34.9 ±5.4		29.6± 6.4		126.2±20.7	
**Occupation**										
Government employee	28.6±4.1	0.54	32.3±4.5	0.000	33.3±5.9	0.000	30.2 ± 5.7	0.01	127.1±20.2	0.000
House wife	29.7±4.9		30.3± 4.5		32.5±5.2		29.1± 5.5		121.6±20.1	
Farmer	28.7±5.1		29.2± 4.9		28.1±6.1		28.1± 7.8		114.1±23.9	
Merchant	29.6±4.4		31.4 ±4.2		31.9±4.7		31.4 ± 4.2		124.3±17.5	
Student	28.7±4.7		28.8±5.7		31.3±6.4		29.2 ± 5.8		118±22.6	
Private employee	27± 4.6		30.1±4.4		31.1± 6		31.2± 8.7		119.4±23.7	
**Monthly household income**										
<750 ETB	28.8± 4.7	0.053	29.3±4.7	0.000	30.2±5.8	0.000	29.9 ± 6.4	0.81	118.2±21.6	0.000
750–1200 ETB	28.5± 4.6		31.1±4.5		32± 5.5		29.6±6.30		121.2±20.9	
>1200 ETB	30.1± 5.0		32.7±4.2		33.9±5.9		30.1 ± 5.4		121.2±20.5	
**Number of HH individuals HH**										
<5	29 ± 4.7	0.91	30.6±4.9	0.83	31.5± 5.9	0.92	30.1 ± 6.1	0.152	121.2±21.6	0.48
>5	28.9± 4.6		30.5±5.8		31.6±5.7		29.1 ±6.4		120.1±22.5	
**Mental health information in the last 1 year**										
Yes	28.3± 4.9	0.004	30.3±4.7	0.29	30.7±6.3	0.002	29.4 ±7.2	0.162	118.7±23.1	0.001
No	29.6± 4.3		30.8±4.9		32.5±5.2		30.2± 4.9		123.1±19.3	
**Ever suffered from any mental illness**										
Yes	29.9 ±5.9	0.25	29.4±5.3	0.10	29.8±5.9	0.03	27.5± 6.8	0.005	116.6±23.9	0.06
No	28.8± 4.5		30.7±4.7		31.8±5.8		30.1 ±6.1		121.4±21.1	
**Ever encounter PWMI**										
Yes	28.9 ±4.5	0.60	30.7±4.8	0.26	31.7±5.7	0.52	29.8± 6.0	0.76	121.1±21	0.73
No	29.1±5		30 ± 4.7		31.2±6.2		30.0 ± 6.7		120.3±22.6	
**Relationship to the PWMI**										
Family	31.4±5.6	0.84	31± 4.9	0.08	31.4±5.7	0.85	30.8± 6.5	0.37	124.6±22.7	0.9
Neighbor	31.3 ± 5.6		31.4± 4.8		31.3±5.6		29.3± 6.3		123.3±22.3	
Friend	32.2± 5.3		30.7± 4.7		32.2±5.3		29.5 ±7.1		124.6±22.4	
No relationship	31.6±6.2		30 ± 4.6		31.6±6.2		29.6±5.8		122.8±22.8	
**Involve in caring for PWMI**										
Yes	28.3±4.9	0.003	30.3±4.7	0.21	30.5±6.3	0.000	29.3 ± 7.3	0.13	118.4±23.2	0.000
No	29.6±4.3		30.9±4.8		32.6±5.2		30.3±4.9		123.4±19.2	
**Threatened or got hurt by PWMI**										
Yes	28.1±4.9	0.000	30.3±4.6	0.28	30.6±6.2	0.001	29.3 ±7.2	0.11	118.3±22.9	0.000
No	29.8±4.3		30.8±4.9		32.5±5.3		30.3 ±4.9		123.4±19.4	
**Witnessed any one being threatened or attacked by PWMI**										
Yes	28.2±4.9	0.001	30.3±4.7	0.28	30.5±6.2	0.000	29.4 ± 7.2	0.10	118.4±23	0.000
No	29.7±4.3		30.8±4.9		32.6±5.3		30.2 ±4.9		123.3±19.4	

### Factors associated with CAMI subscales

**Authoritarianism:** In multiple linear regressions one-way ANOVA and independent t-test showed there was a significant difference in scores of authoritarianism by getting mental health information within the last one year. Those who had mental health information within the last one year had less view of Authoritarianism (β = -0.10, P< 0.05) towards the person with mental illness.

**Benevolence:** Occupation was significantly associated with benevolence. Farmers (β = -0.193, P< 0.05) and students (β = -0.298, P< 0.01) had less scores of benevolence compared to housewives respectively. This implies that both students and farmers were more stigmatizing, in which they have less humanistic and sympathetic view towards people with mental illness than housewives.

**Social Restrictiveness:** Those who were at the level of college or university had 0.29 higher score of social restrictiveness than who were unable to read and write (β = 0.29, P<0.05). Farmers had 0.291 score of social restrictiveness than housewives (β = 0.291, P<0.05). However, those who had mental health information were less likely to be socially restrictive (β = -0.587, P<0.05).

**Community mental health Ideology:** Only age was significantly associated with community mental health ideology. Those who were aged 48 years and above had 0.59 lower score of community mental health ideology (β = -0.59, P< 0.05) compared to those between 18–27 years. This indicates acceptance of community mental health service and integration of PWMI to the community was less among respondents aged 48 years and above (Table 4).

**Table 4:** Multiple linear regression analysis showing significant predictors of CAMI sub scales in Worabe town, Silte Zone, SNNPR, Ethiopia, 2014.

Variables	AU		BE		SR		CMHI	
	Β	CI	Β	CI	Β	CI	β	CI
Age above 48 years							-0.59	(-1.09, -0.08)
College or university			0.01	(-0.32, 0.35)	0.29	(0.059,0.54)*		
Farmer			-0.19	(-0.36, -.03)*	0.291	(0.09, 0.49)*	-0.15	(-0.48, 0.185)
Students			-0.29	(-0.48, -0.12)*	-0.26	(-0.68, 0.17)	-0.11	(-0.47, 0.26)
Income >1200 ETB					-0.31	(-0.60, -0.02)*		
Getting mental health information	-0.10	(-1.11, -0.06)*			-0.59	(-1.13, -0.05)*		

**Note:** Reference categories for the variables listed on the Table 4 are:

Age: 18–27 years

Education: unable to read and write

Occupation: housewife

Income: <750 ETB

* indicates significant at p<0.05

## Discussion

Education was associated with social restrictiveness in a way that those who were at the level of college or university have higher score of social restrictiveness than respondents who were unable to read and write, these grossly show; with better educational status more negative attitude of social restrictiveness. This indicates highly educated people have higher expectations of performance of social responsibility than less educated people might be because they couldn’t trust the mentally ill [12]. When we consider the overall attitude score, however, those with educational status at primary level were less stigmatizing than illiterate ones. This finding is in line with the study conducted in GGFRC [11]. This might be attributed to in fact as people become more educated the chance of getting information on mental health is better.

Occupation was found to be associated with social restrictiveness and benevolent subscales. Farmers were more social restrictive and less benevolent than housewives. Also students had less benevolent attitude than housewives. This implies that farmers generally had more view of considering mentally ill as threat to society but had less sympathetic and humanistic view towards mentally ill than that of house wives. The study found that house wives had more humanistic and sympathetic view towards persons with mental illness. This could be due to that most housewives are expected to be more caring and sympathetic in the community as the way they behave towards their child/children. Also they are less comfortable on avoiding persons with mental illness away from the community [11]. However, studies conducted in Nigeria and India showed occupation was not associated with any of the subscales and the difference might be attributed to the difference in study setting and sample size [17,23].

Residents with income more than 1200 ETB were found to be less socially restrictive compared with income less than 750 ETB. This indicates as income of the individuals increase, they tend to be less socially restrictive. But the study conducted in Toronto reported that household income found to have no association to any of the subscale [13]. The variation might be due to difference in social, cultural and economic characteristics. This finding is consistent with the study conducted in GGFRC [11].

Respondents who got mental health information within the last 1 year independently were less socially restrictive and less authoritarian. This might be because, it is expected that those who have mental health information could have understanding on the nature of mental illness which may result in accepting PWMI and avoiding inferiority view towards PWMI. This can be explained by 50.3% of respondents reported that they had information about mental illness. From FGD, 42 years old male participant said *“…in Worabe town people are afraid to marry someone from a family where mental illness exists; in case the new children get ill*. *But they are mistaken*. *For example*, *the husband of my daughter had been psychiatric patient for many years in Amanuel Hospital but after he started treatment he gets well*, *now they have 2 children and all are ok*, *am also happy with their marriage*.*”*

But mental health information was not associated with any of the subscales in studies done in GGFRC, Nigeria and India. This difference might be due to that urban and rural residents are included in GGFRC study [11], subjects were medical workers only with little occupational variation in Nigeria[23] and few study subjects (100) in India [17].

Residents aged greater than 48 years of age had less community mental health ideology when compared to those whose age is between 18–27 years. This implies the acceptance of mental health services and the integration of persons with mental illness in the community get decrease with age 48 years and above. This might be because most aged community members though traditional medicine is preferable than modern medicine to treat mental illness since 56.1% of respondents had negative view on the provision of mental health services through community based facilities. This may have an implication in which it can be a big obstacle while attempting to run mental health services integration program to the community health services.

A 66 years old male participant also said *“…he or she (mentally ill) is a mad person*, *so what is he or she doing in the community*…*Even the government should build health institution in restricted area to protect the community because they are dangerous*, *unless it will interfere with our daily life when we became aged and also they may harm our young children*.*”*

But a study done in Taiwan showed as age increase the tendency of positive attitude also increases [12], where as a study conducted in GGFRC showed, for a unit increase in age the level of negative attitude decreases [11].

The variation might be due difference in study setting and method of data collection.

## Conclusion and Recommendation

Negative attitude towards people with mental illness is widely held across all the subscales and implies that residents of Worabe town were more socially restrictive and were poorly oriented to community mental health services. Despite the fact revealing lower proportion of study participants had mental health information, health service institutions had minor contribution as a source of information compared to other sources.

Mental health information was the most important variable associated with Authoritarianism, and Social Restrictiveness. High level of education was related with more social restrictiveness in the integration of persons with mental illness in the community.

There is a need to develop strategies to aware the public regarding the nature of mental illness to promote acceptance of PWMI by the community. It will be great to facilitate launching of the rehabilitation center in the town to decrease social restrictive view of the community as well as to give better treatment and reduce aggression. Conducting further research focusing on the relationship between knowledge and attitude towards mental illness is important to address knowledge related factors associated with negative attitude towards PWMI.

## Supporting Information

S1 Table Socio Demographic characteristics of respondents of Worabe town, Silte zone, SNNPR, Ethiopia, 2014. (DOCX)
