# Supplementary material for: Community Attitude and Associated Factors towards People with Mental Illness among Residents of Worabe Town, Silte Zone, Southern Nation’s Nationalities and People’s Region, Ethiopia
Source: PLoS One. 2016 Mar 1;11(3):e0149429. doi: 10.1371/journal.pone.0149429 (PMC4773224; doi:10.1371/journal.pone.0149429)
Supplement: S1 Table — (DOCX) [file pone.0149429.s001.docx]

S1 Table. Socio Demographic characteristics of respondents of Worabe town, Silte zone, SNNPR, Ethiopia, 2014

| **Variables** | **Frequency** | |
| --- | --- | --- |
|  | **N** | **%** |
| **Sex** Male | 203 | 49.8 |
| Female | 205 | 50.2 |
| **Marital status** Married | 277 | 67.9 |
| Single | 113 | 27.7 |
| Divorced | 11 | 2.7 |
| Widowed | 7 | 1.7 |
| **Ethnicity** Silte | 333 | 81.6 |
| Gurage | 22 | 5.4 |
| Oromo | 13 | 3.2 |
| Amhara | 13 | 3.2 |
| Tigre | 5 | 1.2 |
| Others* | 22 | 5.4 |
| **Religion** Muslim | 318 | 77.9 |
| Orthodox | 41 | 10.0 |
| Protestant | 21 | 5.1 |
| Catholic | 9 | 2.2 |
| Others****** | 19 | 4.7 |
| **Educational level**  Primary | 127 | 31.1 |
| Secondary | 91 | 22.3 |
| Unable to read and write | 83 | 20.3 |
| Able to read and write only | 58 | 14.2 |
| College or university | 49 | 12.0 |
| **Occupation** Housewife | 86 | 21.1 |
| Merchant | 86 | 21.1 |
| Civil servant | 83 | 20.3 |
| Farmer | 70 | 17.2 |
| Student | 47 | 11.5 |
| Private employee | 35 | 8.6 |
| **Income** <750 ETB | 191 | 46.8 |
| 750-1200 ETB | 139 | 34.1 |
| >1200 ETB | 78 | 19.1 |

**Note**: *Hadiya, Alaba, Wolaita **Jehovah
